# Robot-assisted radical nephrectomy for primary renal mesenchymal chondrosarcoma: case report and literature review

**DOI:** 10.1080/0886022X.2019.1583111

**Published:** 2019-03-26

**Authors:** Wen Deng, Jinxiu Zhou, Xiaoqiang Liu, Luyao Chen, Guanghua Guo, Bin Fu

**Affiliations:** a Department of Urology, The First Affiliated Hospital of Nanchang University, Nanchang, Jiangxi Province, China;; b Department of Burns, The First Affiliated Hospital of Nanchang University, Nanchang, Jiangxi Province, China

**Keywords:** Kidney, mesenchymal, chondrosarcoma, treatment, robot-assisted surgery

## Abstract

As an extremely rare malignant neoplasm, only 12 mesenchymal chondrosarcoma (MC) arising in kidney have been reported to date. Herein, we reported a case of primary renal MC resected with robot assistance, which has not been reported before. According to the cases reported in English literature, we analyzed the characteristics of this rare malignancy and systematically review its treatment.

## Introduction

Mesenchymal chondrosarcoma (MC), a specific histological pattern consisting of highly undifferentiated small cells and islands of well-differentiated hyaline cartilage, was initially described by Lichtenstein and Bernstein in 1959 [1–3]. While mostly derived from the osseous tissue, only 20–33% of these uncommon tumors arise in extraskeletal sites [4], including the head and neck region, followed by lower extremity, the trunk, and the retroperitoneum [3,5]. Only exceedingly limited cases originating from kidney have been reported in English literature [1–3,5–13].

Here, we report a case of primary renal MC resected with robotic assistance, which has not been reported before to our knowledge. Along with those reported renal MC cases, the features and management of this rare neoplasm will be discussed.

## Case presentation

**Figure 1.**
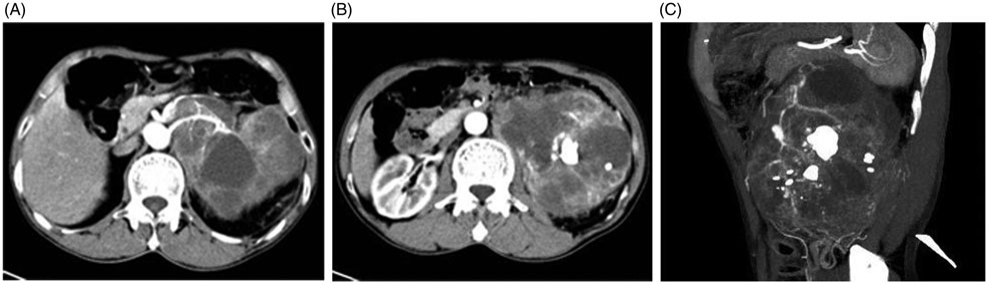
(A) Shows a heterogeneous lobulated mass with expanded renal pelvis, and a bar filling-defect in left renal vein. (B and C) Show calcifications occupying the expanded renal pelvis.

A 62-year-old man, with no significant medical history, was hospitalized with left loin pain and intermittent gross hematuria. Nothing except for mild costovertebral angle tenderness was found abnormal on routine physical examination. The laboratory tests including hematologic studies and urinalysis are shown in Table 1. Abdominal contrast-enhanced CT scan revealed a 14 cm × 11 cm × 8 cm heterogeneous lobulated mass, which involved most of the left renal parenchyma, with the calcification foci and cystic spaces. Multiple patchy dense calcifications occupying the expanded renal pelvis and bar filling defect in left renal vein were also detected in the CT scan (Figure 1). A 0.7 cm lung nodule was identified at the left upper lobe on the chest X-ray. In a bone scan, nuclide was distributed evenly and meristicly over the body except for the 7th thoracic vertebra, which was considered as a metastatic lesion.

**Table 1.** Results of the preoperative laboratory findings.

Hematologic studies			
Hemoglobin	98 g/L	Serum total protein	61.1 g/L
White blood cell	8.77 × 10^9^/L	Albumin	35.3 g/L
Neutrophil	75.8%	Globulin	25.8 g/L
Lymphocyte	16.6%	ALT	9 U/L
Platelet	323 × 10^9^/L	AST	23 U/L
Blood urea nitrogen	7.7 mmol/L	Fasting plasma glucose	4.09 mmol/L
Creatinine	119.0 µmol/L	Procalcitonin	0.34 ng/mL
Na	140 mmol/L	K	4.15 mmol/L
ESR	65 mm/h		
*Urinalysis*			
Red blood cell	80 cell/L	White blood cell	0 cell/L
Specific gravity	1.020		

ESR: erythrocyte sedimentation rate; ALT: alanine aminotransferase; AST: aspartate aminotransferase.

With a clinical diagnosis of left renal cell carcinoma with invasion into renal vein and metastasis to the left lung and 7th thoracic vertebra, the patient underwent robot-assisted left radical nephrectomy with renal vein thrombectomy and lymph node dissection after renal arterial embolization.

**Figure 2.**
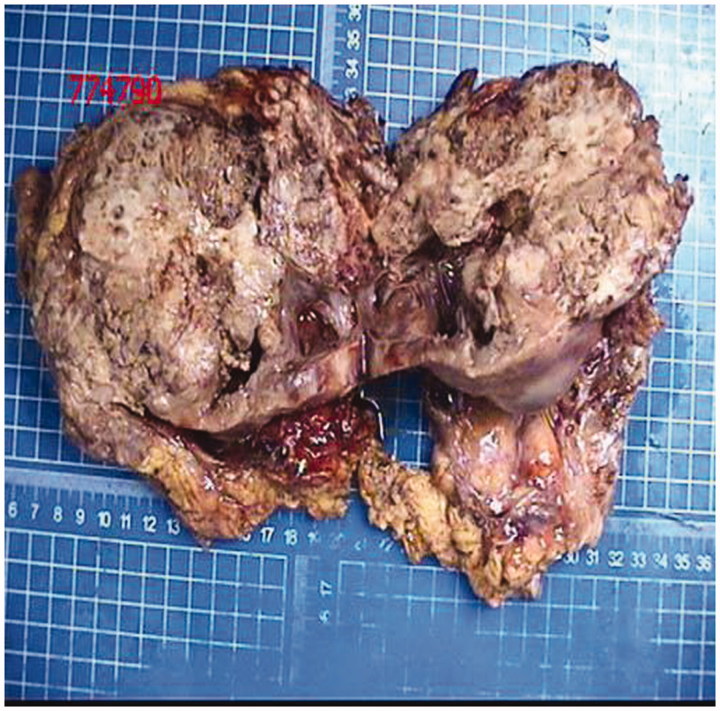
Bivalved gross specimen shows a grayish, fleshy appearance and areas of hemorrhage, necrosis, and calcifications.

**Figure 3.**
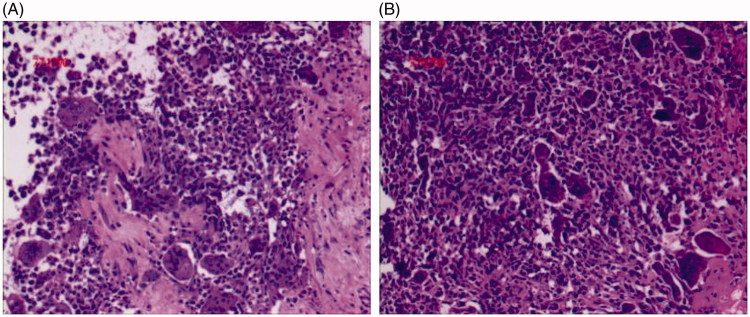
Pathological examination shows the tumor comprised of round or elliptical undifferentiated cells and abundant islands of well differentiated cartilage (hematoxylin and eosin staining; magnification, ×200).

**Figure 4.**
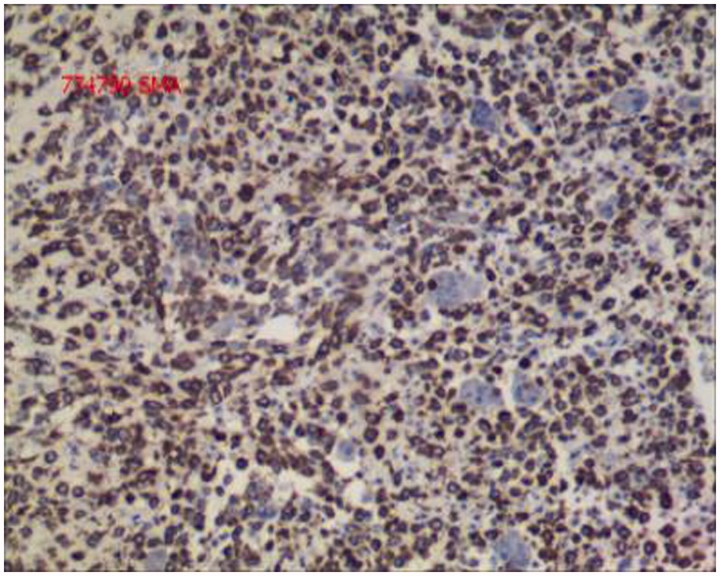
Immunohistochemical staining of the specimen revealing positive staining in the cells for SMA (SMA staining; magnification, ×200).

The gross specimen of the removed kidney measured 16 cm × 15 cm × 9.5 cm (Figure 2). The cut surface in renal parenchyma had shown a huge growth extending to renal pelvis and bulging into the perirenal fat. The cut surface also had a grayish, fleshy appearance and contained areas of hemorrhage, necrosis, and calcifications. Microscopically, the huge tumor comprised diffusely distributed sheets of round or elliptical undifferentiated cells of varying size and abundant islands of well-differentiated cartilage (Figure 3). Tumor necrosis was palpable in some areas. Immunohistochemical (IHC) staining was revealed as follows: S100 (cartilage)+, SMA (+) (Figure 4), Vim (+), Bcl-2 (+), CD68 (+), CD99 (+), Ki-67 (40%+), CK (−), CR (+/−), Des (−), EMA (−), WT-1 (−), CD34 (vessel+), HMB45 (−), and Melan-A (−). The tumor was perfectly resected with wide negative surgical margins, and the tumor thrombus was confirmed to be malignant. Only one renal hilar lymph node was positive among all four renal hilar lymph nodes and six retroperitoneal lymph nodes. A diagnosis of primary renal MC was made according to these pathological results.

During the follow-up, the patient presented severe headache caused by skull metastasis after postoperative 2 weeks. Considering the patient’s progressively weak condition after surgery, supportive care and pain management were pursued until the patient died of widespread metastases at postoperative 1 month, and no adjuvant chemotherapy or radiotherapy was adopted.

## Literature review

A literature search was performed in December 2018 without restrictions to regions. The primary source was the electronic PubMed database. The following terms and their combinations were searched: (‘renal’ OR ‘kidney’ [Title/Abstract]) and (‘chondrosarcoma’ [Title/Abstract]) and (‘mesenchymal’ [Title/Abstract]). Our computer search was also supplemented with manual searches of reference lists of all retrieved articles. About 12 cases of renal MC have been previously reported in English literature (Table 2) [1–3,5–13].

**Table 2.** Summary of primary renal mesenchymal chondrosarcoma cases.

Study	Age, years	Sex	Location	Presentation	Imaging features	Tumor size^c^, cm	Metastasis	IHC	Adjuvant radiotherapy	Chemotherapy	Postoperative outcome
Malhotra (1984) [1]	27	M	L, LP	GH, FP	TC^a^	9	Right parietal region PO 28m	Na	4000r, 15T	ABCD	Multiple metastasis after 28 months
Karanauskas (1991) [13]	15	M	R	Axilla metastasis	TC^a^	Na	Lung, right axilla	Na	Na	Na	Na
Gomez-B (2001) [12]	52	F	R, UP	GH	TC^a^	8	No	Na	No	No	Normal in 12 months
Kaneko (2006) [11]	61	F	R	Incidental mass	TC^a^	2.5	No	vim, S	No	no	Normal in 6 years
Xu (2012) [2]	64	M	L, UP	Loin pain, GH	LS on T1^b^	11	Widespread PO 2m	vim, S	No	No	Died 2 months later
Tyagi (2014) [9]	22	F	R, MP	FP, GH, HF	HM^a^	6	Lung	vim, S	No	CE	Stable disease in 21 weeks
Chen (2015) [8]	17	M	R, UP	FP, HF	HM^a^	10	No	CD^┬^68/57/99, myo	No	CD	Stable disease in 10 months
Rothberg (2015) [3]	16	F	L, LP	AP, headache	HM, TC^a^	15.2	Lung, Cervical spine	Na	Na	Na	Na
Pani (2017) [7]	24	M	R, UP	FP	HM, TC^a^	8.5	No	Na	No	DI	Symptom-free in 6 months
Salehipour (2017) [6]	22	M	R, MP	FP	HM, TC^a^	9	No	vim, bcl2, CD*99	Na	Na	Na
Gherman (2014) [10]	67	M	L	Lumbar pain, GH	HM, TC^a^	30	Lung PO 6m	Na	No	CD	Died 9 months after surgery
Valente (2018) [5]	35	M	L	FP, GH	TC^a^	20	No	vim, CD^c^99, S	No	No	Normal in 18 months
The present case	62	M	L	Loin pain, GH	HM, TC^a^	14	Lung, thoracic vertebra	vim, CD*99			
S, SMA	No	no	Died 1 month after surgery								

IHC: immunohistochemical; M: male; F: female; L: left; R: right; UP: upper pole; MP: mid-pole; LP: lower pole; FP: flank pain; GH: gross hematuria; HF: high fever; AP: abdominal pain; TC: tumor calcification; LS: low signal mass; HM: heterogeneous mass; PO: postoperative; na: not available; vim: vimentin; S: S-100 protein; CD*: cluster of differentiation; myo: myogenin; SMA: smooth muscle actin; T: treatment; A: adriamycin; C: cytoxan; B: bleomycin; D: doxorubicin; I: ifosfamide; E: epirubicin.

a On CT image.

b On MRI image.

c Maximal tumor diameter of specimen (cm).

## Discussion

MC comprises 2–9% of all chondrosarcomas [14], and most of them originate in the bone [6]. MC rarely occurs in the kidney, and only 12 cases have been reported in English literature [1–3,5–13].

A peripheral distribution of MC is found in patients aged 50 years and older, while the peak incidence for MC is in the 2nd–3rd decade of life [15–17]. As shown in Table 2, seven patients are aged 10–30 [1,3,6–9,13] while four cases [2,10–12] and one of our cases are aged ≥50, which was consistent with the former conclusion. MC occurs in both genders equally [16], while extraskeletal MC has a mild female preponderance [4]. However, the occurrence of renal MC seems to a masculine orientation (9/13) [1,2,5–8,10,13], which needs other cases to confirm further.

As shown in Table 2, similar to renal clear cell carcinoma, renal MC mostly (six of eight renal MCs of whom the information of locality was available) occupied the upper or lower pole of the affected kidney [1–3,7,8,12]. Most patients [1–3,5–10,12] with renal MC have flank pain, gross hematuria, or high fever, and the minority complained of symptoms caused by metastatic lesions [13]. Extraskeletal MC has a strong tendency toward recurrence and late metastases [4,16,18], thereby affecting survival negatively [14,16]. The most potential metastatic foci are lung, followed by skeleton and lymph nodes [3,5,9,10,13,19] (the fifth one was our case)/13 and 3 [1,3] (the third one was our case)/13 cases in Table 2 had metastasized to lung and bone, respectively.

The presence of calcification, the key to an imaging diagnosis of MC [18], was observed in up to 67% of the chondrosarcoma cases according to CT scans [20]. The difference in the peripheral distribution and central distribution of the calcification on CT scans has been reported in several studies [17,20]. The characteristic ring-and-arc mineralization has definite value in qualitative diagnosis [17]. As to renal MC, a total of 10 (within our owe case) out of 12 cases providing information about CT imaging revealed calcifications on CT scans [1,3,5–7,10–13], while the remaining two patients revealed a heterogeneous mass without calcification [8,9]. On the MR imaging result of the extraskeletal MC cases, equal or low and high-low mixed signal intensity is often shown in T1W1 and T2WI, respectively [17]. A diffused heterogeneous enhancement or nodular enhancement in both calcified and non-calcified areas on enhanced MR imaging is considered as an important diagnostic sign for extraskeletal MC [17,21]. The case reported by Xu et al. [2] showed a low signal on both T1WI and T2WI on MR imaging.

The coexistence of benign tumors’ features and imaging performance similar to other malignant soft tissue tumors in extraskeletal MCs made it hard to differentiate from other tumors [17]. The diagnosis of MC remains dependent on pathological examination, which exhibits a characteristic pattern composed of sheets undifferentiated round, oval, or spindle-shaped cells and islands of well-differentiated hyaline cartilage [22,23]. On immunohistochemistry, the areas of well-differentiated cartilage often express non-specific markers of cartilaginous differentiation, such as S-100 protein and CD57 (Leu 7) [22], and relatively specific markers, such as SOX9, a marker that simultaneously shows nuclear positivity in undifferentiated mesenchymal cells [4]. Meanwhile, CD99 expression is common in undifferentiated cell sheets [4,22]. The positive staining for Sox9 along with negative staining for FLI-1 aids in distinguishing MC from Ewing sarcoma [16]. Immunohistochemical staining was performed in seven cases (the seventh case was our case) of all included renal MC patients [2,5,6,8,9,11], in five cases [2,5,9,11] (the fifth case was our case), the cells within the cartilaginous areas are positive for S-100, while CD99 and Vim expressed by the poor-differentiated tumor cells are positive in four [5,6,8] (the fourth case was our case) and six patients [2,5,6,9,11] (the sixth case was our case), respectively.

The treatment of primary MC has no widely accepted guidelines due to its rarity and the lack of appropriate studies and clinical trials to assess the best management [10]. Increasing studies have recommended surgical resection with wide margins as the gold standard to treat extraskeletal MC, but the roles of radiotherapy and chemotherapy remain controversial [5–8]. Da Vinci robotic surgery has never been performed to treat renal MC before our case presentation, in which the renal MC was perfectly resected in spite of the large tumor size.

Many studies have found that adjuvant radiotherapy for patients with MC plays a role in local tumor control, but the association with overall, disease-free, or metastasis-free survival is insignificant [19,24], that is, patients have the same risk of metastasis or death although they avoided local recurrence.

A consensus on the doxorubicin-based chemotherapy regimens as the adjuvant therapy of MC has been reached [6,7,19]. However, controversy still exists. Kawaguchi et al. [24] found that adjuvant doxorubicin-based chemotherapy did not significantly relate to disease-free survival in the analysis of 19 cases. Meanwhile, Cesari et al. [25] suggested the addition of chemotherapy after complete surgical remission is beneficial in terms of disease-free survival in MC treatment. Gherman et al. [10] recommended neoadjuvant chemotherapy followed by a wide surgical resection as the best management of MC and attempted to establish a standard of care for similar patients. More renal MC cases with extensive follow-up are needed to confirm better treatment.

Many new treatment strategies have been introduced. Shakked et al. [26] introduced therapies targeting the platelet-derived growth factor receptor α proliferation pathway may be beneficial in treating MC. de Jong et al. [27] reported that the inhibition of Bcl-2 family members sensitizes MC to conventional chemotherapy, which provides new methods for treating MC. Many studies have implied that HEY1-NCOA2 rearrangement is not only a diagnostic marker but also a potential therapeutic promoter for MC [28,29]. However, all these new opinions need further verification.

Prognosis of MC differs with the variation of the site of tumor origin [14,30]. The 10-year survival rates for patients with MC vary from 33% to 67% in the literature [16,24,30]. The patient with the smallest tumor burden remained disease-free after 6 years [11], which indicated the importance of early diagnosis and treatment. Close follow-up is extremely important after surgery.
